# Contrasting seasonal patterns and factors regulating biocrust N_2_-fixation in two Florida agroecosystems

**DOI:** 10.3389/fmicb.2022.892266

**Published:** 2022-08-03

**Authors:** Kira Sorochkina, Sarah L. Strauss, Patrick W. Inglett

**Affiliations:** ^1^Department of Soil and Water Sciences, University of Florida, Gainesville, FL, United States; ^2^Southwest Florida Research and Education Center, University of Florida, Immokalee, FL, United States

**Keywords:** nitrogenase (acetylene reducing) activity, nitrogen fixation, biocrust, vineyard, citrus orchard, agroecosystem

## Abstract

Biocrusts are communities of microorganisms within the top centimeter of soil, often dominated by phototrophic dinitrogen-fixing (N_2_-fixing) organisms. They are common globally in arid ecosystems and have recently been identified in agroecosystems. However, unlike natural ecosystem biocrusts, agroecosystem biocrusts receive regular fertilizer and irrigation inputs. These inputs could influence seasonal biocrust N_2_-fixation and their relationship with soil nutrients in perennial agroecosystems, which is of particular interest given crop management requirements. In this study, biocrust and adjacent bare soil N_2_-fixation activity was measured in the field during the summer, fall, spring, and winter seasons in a Florida citrus orchard and vineyard using both acetylene reduction assays and ^15^N_2_ incubations. Samples were analyzed for microbial and extractable carbon (MBC, EC), nitrogen (MBN, EN), and phosphorus (MBP, EP). In both agroecosystems, biocrusts had greater microbial biomass and extractable nutrients compared to bare soil. The citrus and grape biocrusts were both actively fixing N_2_, despite crop fertilization, with rates similar to those found in natural arid and mesic systems, from 0.1 to 142 nmol of C_2_H_4_ g^–1^ of biocrust dry weight h^–1^ (equivalent to 1–401 μmol m^–2^h^–1^). Lower soil temperatures and higher EC:EN ratios were associated with higher N_2_-fixation rates in citrus biocrusts, while higher soil moisture and higher EP were associated with higher N_2_-fixation rates in grape biocrusts. The N_2_-fixation activity of these agroecosystem biocrusts indicates the possibility of biocrusts to enhance N cycling in perennial agroecosystems, with potential benefits for crop production.

## Introduction

Biological soil crusts (biocrusts) are communities of organisms on soil surfaces and often include diazotrophic organisms such as lichens (Kuske et al., 2012), cyanobacteria (Yeager et al., 2007), and heterotrophic bacteria (da Rocha et al., 2015), in addition to other non-diazotrophs such as archaea (Zhao et al., 2020), green algae, fungi (Bates et al., 2010), and bryophytes (Seitz et al., 2017). Biocrusts occur on all continents (Colesie et al., 2016), and are adapted to higher light exposure (Garcia-Pichel and Castenholz, 1991), lower water availability (Garcia-Pichel and Pringault, 2001), and cycles of water saturation and drying typical in deserts (Rajeev et al., 2013). However, biocrusts are not restricted to arid conditions and have also been identified in mesic ecosystems (Veluci et al., 2006; Seitz et al., 2017), where they experience temporary dry conditions, similar to their arid counterparts, which require adaptations to desiccation and high light exposure (Colesie et al., 2016). Recently, biocrusts have also been identified in managed mesic ecosystems, specifically perennial Florida agroecosystems (Nevins et al., 2020, 2022) where they occur in unshaded areas between trees (e.g., citrus orchards) and grape vines. However, unlike the natural ecosystems where biocrusts have been well studied, considerably less is known about the processes and functions of biocrusts in agroecosystems.

Biological nitrogen fixation (N_2_-fixation) is one of the key biocrust processes, and it is estimated that biocrusts contribute approximately half of the total N fixed in arid lands (Elbert et al., 2012), with rates between 0.08 and 10 kg N ha^–1^ year^–1^ (Malam Issa et al., 2001; Belnap, 2002; Billings et al., 2003; Russow et al., 2005; Housman et al., 2006; Holst et al., 2009). Similarly, mesic biocrusts fix 1.3 kg N ha^–1^ year^–1^ in a temperate savannah (Veluci et al., 2006), 5.2 kg N ha^–1^ year^–1^ in a seasonally flooded savannah (Williams et al., 2018), and 4 kg N ha^–1^ year^–1^ in the seasonally flooded Everglades (Liao and Inglett, 2014).

In agroecosystem biocrusts, the possibility of naturally occurring N_2_-fixation is of particular interest due to the fertilizer requirements for perennial crops. However, agroecosystem biocrust N_2_-fixation rates may differ from those of natural ecosystems due to the influence of N and phosphorus (P) fertilization applications. In particular, fertilizer N could inhibit biocrust N_2_-fixation, as shown both in field-collected biocrusts and multi-species laboratory cultures. For example, N additions mimicking atmospheric deposition significantly reduced N_2_-fixation rates in arid light and dark biocrusts (Belnap et al., 2008). In addition, 25 days of exposure to 55 lbs N acre^–1^ reduced N_2_-fixation of agroecosystem biocrust cell cultures by 80% (Peng and Bruns, 2019). Based on this N application rate with the assumption that 25 days of constant fertilizer exposure are equivalent to a yearly fertilization rate, it is common to split liquid fertilization in citrus into 26 biweekly doses. Such a fertilization rate would be within the lower range for a 1 to 3-year-old citrus in the field: 25–200 lbs acre^–1^ (Obreza and Morgan, 2020). Therefore, it is expected that agroecosystem biocrusts will have drastically reduced N_2_-fixation rates compared to their natural ecosystem counterparts.

In contrast to N, however, P addition could stimulate N_2_-fixation. Phosphorus addition to a P-limited environment enhanced N_2_-fixation activity and labile P concentration in non-biocrust soils of prairie and tropical rainforest (Reed et al., 2007a,b). Furthermore, the balance of P and N availability dictated N_2_-fixation rates of wetland biocrusts (Liao and Inglett, 2014). Biocrust N_2_-fixation in a P amended agroecosystem could therefore be higher than in natural ecosystem biocrusts.

While N_2_-fixation positively responded to increases in moisture in arid ecosystems (Zhao et al., 2010; Caputa et al., 2013) and seasonally flooded biocrusts of restored wetlands (Liao and Inglett, 2012, 2014), agroecosystem biocrust N_2_-fixation rates are not expected to vary as strongly seasons because of consistent moisture provided by irrigation. While higher temperature ranges are associated with higher N_2_-fixation rates in natural ecosystem biocrusts from regions with temperatures ranging from below 0 to 30°C (Zhao et al., 2010; Caputa et al., 2013), higher N_2_-fixation rates are not expected in agroecosystem biocrusts during the warmer seasons of fall (17–28°C), spring (16–26°C), and summer (25–29°C) than in the cooler winter (10–22°C) due to their narrow temperature range.^1^

Biocrust N_2_-fixation activities have not been examined in perennial agroecosystems, and the influence of consistent N and P fertilization and irrigation on agroecosystem biocrust N_2_-fixation activities is unknown. Therefore, we conducted a year-long field study of agroecosystem biocrusts and bare soil controls to quantify seasonal N_2_-fixation rates, compare biocrust and bare soil nutrient concentrations, and identify the relationships between biocrust N_2_-fixation activity, soil nutrients, and environmental variables. We chose two agroecosystems with biocrusts (a vineyard and a citrus orchard) that had similar climatic conditions but differed in fertilization and irrigation management. We hypothesized that due to limited seasonal temperature change and consistent water input through irrigation, N_2_-fixation rates of these agroecosystem biocrusts would not have a seasonal pattern. However, based on differences in crop management, we hypothesized that the availability of N and P fertilizer would regulate biocrust N_2_-fixation patterns and relationships with biocrust nutrient concentrations more strongly than soil temperature and soil moisture.

## Materials and methods

### Site and plot selection

This study was conducted in a subtropical climate receiving 813–929 mm total precipitation during the study period from August 2019 to May 2020 with mean air temperatures ranging from 21°C to 22°C.^1^ Agroecosystem biocrusts were assessed in two perennial crops: 2-month-old *Vitis rotundifolia* (muscadine grape) located at the University of Florida Plant Science Research and Education Unit in Citra, Florida (referred to as ‘Grape’, 29.407195, –82.139980) and a 2-year-old *Citrus sinensis* (orange) orchard located at the University of Florida Citrus Research and Education Center in Lake Alfred, Florida (referred to as ‘Citrus’, 28.115496, –81.713458). Soils of both sites were classified as excessively drained Entisols of the Candler series, sandy soil formed from eolian and loamy marine deposits with 6.0–6.5 pH as measured by Jalpa et al. (2020) and Nevins et al. (2020). Citrus was irrigated daily through micro-sprinklers, while Grape was irrigated daily through a drip system. Each Grapevine received at least 3.8 L of water per day from May to June, and then this amount was reduced by half during the other months. Each Citrus tree received approximately 34 L of water per day, and this amount was reduced by half during the winter. Grape had 11 N kg ha^–1^ 10–10–10 NPK granulated fertilizer applied in June 2019, July 2019, and March 2020, while Citrus was fertigated (5–0–7 NPK or 7–2–7 NPK) weekly and received 29 N kg ha^–1^ 12–4–8 NPK controlled release fertilizer in July 2019 (Supplementary Table 1).

Samples were collected at Citrus in September 2019 (Summer), November 2019 (Fall), January 2020 (Winter), and May 2020 (Spring); and at Grape in August 2019 (Summer), October 2019 (Fall), January 2020 (Winter), and May 2020 (Spring). Samples were collected from six plots at each location. Each plot was randomly located on either side of the crop trunk or vine and contained an intact biocrust and adjacent bare soil (no more than 10 cm away from the biocrust) located within the crop row (Supplementary Figure 1). There were minimal weeds in each plot due to herbicide control using glyphosate, and plots were located 122 cm away from the trunk or vine.

Biocrusts and bare soils were randomly sampled at each plot and ranged in area from 1045 cm^2^ up to 6427 cm^2^. Plots were at least 2 m apart from each other within a site. After each sampling point, the sample collection plot locations were shifted to the nearest intact biocrusts (no more than 2 m away from the original plot location) because not enough material remained for repeated sampling.

### Qualitative biocrust characterization

Biocrusts were identified by field observations and referencing the visual development scale (Belnap et al., 2008). The bare soil for each plot had no visible surface roughness or darkening (Belnap et al., 2008). To further characterize biocrusts, two replicates of biocrusts from sampling times during which they exhibited the highest N_2_-fixation rates from both sites (Grape: August 2019; Citrus: May 2020) were examined for the presence of cyanobacteria and algae using an inverted microscope, Nikon Eclipse Ti2 (Nikon Instrument Inc., Japan).

### Field environmental parameters

Soil surface temperature and light intensity were measured at each plot (*n* = 6) when samples were collected. Temperatures were measured at the surface without plant shading using a thermocouple attached to a DIGI-SENSE 20250-02 temperature meter (Cole Parmer, Vernon Hills, IL, United States). Light intensity measurements were recorded using a LI-250A light meter (LI-COR Biosciences, Lincoln, NE United States). Additional data about precipitation and solar irradiance were obtained from the Florida Automated Weather Network^1^ (Supplementary Table 1).

### Sample collection

Three subreplicates each of biocrust and adjacent bare soil were collected intact from each plot to 0.5 cm depth using a 3 cm diameter corer (Supplementary Figure 1). Cores were placed in airtight jars for N_2_-fixation assays and subsequent measurements of soil moisture, microbial biomass, and extractable nutrients. Additional subreplicates for ^15^N enrichment incubations and analysis were collected during the summer season in Grape (biocrusts: *n* = 3, bare soils: *n* = 3) and during the fall season in Citrus (biocrusts: *n* = 2). Bare soil samples from Citrus were not collected for ^15^N^2^ enrichment incubations.

Both sites were irrigated daily in the morning, including the morning before sample collection. However, at the January and May 2020 sample collections, the Grape soil was very dry, therefore, on these dates, the soil was saturated with deionized water before soil core collection. No deionized water was added during other collection times.

### Field N_2_-fixation rate measurements

Biocrust and bare soil N_2_-fixation was measured under field conditions immediately after collection using an adapted version of the acetylene reduction assay (ARA) (Stewart and Bergersen, 1980; Inglett, 2013). Measurements were made using a 2-h field incubation in airtight 138 mL glass jars with 10% acetylene headspace. Non-acetylated sample blanks were simultaneously incubated for biocrusts and bare soils. Intact soil and biocrust core samples were placed on the inside lid of inverted glass jars to allow for increased ambient light access to potentially N_2_-fixing phototrophic organisms (Supplementary Figure 2). The lids had a butyl rubber septum installed into a drilled hole for gas injections. To maintain field soil temperatures and prevent overheating, jars were incubated in a shallow water bath monitored with a thermocouple. After incubation, a 5 mL headspace gas sample was collected into a 3.5 mL exetainer after vigorously shaking each jar for 4 s. The jars were then opened and aerated in the field for at least 10 min before closing and storing them at 4°C for transport to the University of Florida Wetlands Biogeochemistry Laboratory in Gainesville, FL. Gas samples from ARA measurements were stored at 25°C for later ethylene analysis by gas chromatography.

### Acetylene reduction assay calibration incubations

To identify the conversion ratio from acetylene reduction to N_2_ fixed, separate incubations with ^15^N_2_ gas were conducted simultaneously with ARA on two biocrust replicates from Citrus in the fall, three biocrust replicates from Grape in the summer, and three bare soil replicates from Grape in the summer season following the method of Inglett (2013). These incubations were identical to those of ARA, but without injection of acetylene. Briefly, 20 mL of 98% ^15^N_2_ was injected into the jar headspace of samples and into three empty jars. Gas samples were collected from these jars after a 2-h incubation to determine the headspace ^15^N_2_ concentration. The jars of ^15^N_2_ enriched samples were then opened and aerated for 10 min before being placed on ice to stop the incubation. These samples were used for N isotopic determination. Acetylated samples from the same plots where ^15^N_2_-incubated samples were collected served as unenriched controls. ^15^N_2_-enriched biocrust subreplicates from ^15^N_2_-enriched jars and non-enriched control biocrust subreplicates from acetylated jars were separated from loose soil particles with a 0.5 mm sieve. The three sieved enriched biocrust, non-enriched biocrusts, enriched bare soil, and unenriched bare soil subreplicates from each plot were pooled, homogenized, and dried at 70°C.

### Laboratory analysis

Biocrust samples from acetylated jars were separated from loose soil particles with a 0.5 mm sieve. The three sieved biocrust and bare soil subreplicates from each plot were pooled, homogenized, and then subsampled for microbial biomass, extractable nutrients, and moisture determination (*n* = 6). The soil moisture content of biocrusts and bare soils was determined gravimetrically after drying in the laboratory oven for 72 h at 70°C to avoid additional mass loss due to organic matter volatilization and to allow for subsequent N analyses (Susha Lekshmi et al., 2014). Biocrust and bare soil moisture measurements from each plot were averaged together for soil moisture comparison between seasons across sites because no significant difference was detected between biocrust and bare soil moisture (*n* = 12). However, only biocrust soil moistures were averaged together for principal component analysis (PCA).

Microbial biomass C (MBC), N (MBN), and P (MBP) were measured using the fumigation extraction approach (Liao et al., 2014). Briefly, 1 g of sample was incubated for 24 h either in the presence of chloroform (fumigated) or without exposure to chloroform (non-fumigated) at room temperature and then extracted with 0.5 M potassium sulfate (MBC and MBN) or 0.5 M sodium bicarbonate (MBP). The C and N extracts were analyzed using a Shimadzu TOC-5050 (Japan, Tokyo) analyzer with an N module. The P extracts were digested with sulfuric acid and potassium persulfate, resuspended in double deionized water, and analyzed on the Shimadzu UV-160 spectrophotometer (Kyoto, Japan) using the molybdenum blue method (EPA Method 365.3).

Microbial biomass C, MBN, and MBP were determined by calculating the differences between fumigated and non-fumigated sample pairs with 0.37 adjustment factor (extraction efficiency) for MBC, 0.54 for MBN, and no adjustment factor for MBP following McLaughlin et al. (1986). Non-fumigated C and N fractions were quantified as extractable C (EC) and extractable N (EN), while unfumigated P extract was quantified as extractable P (EP) (Olsen et al., 1954). EC and EN are equivalent to KCl-extractable C and N, respectively, while EP is considered to contain available P in both organic and inorganic forms (Liao et al., 2014).

Gas samples from ARA measurements were stored at 25°C and analyzed for ethylene within 2 weeks of collection using a Shimadzu GC-8A gas chromatograph equipped with a flame ionization detector (FID) and HayeSep N column (2 m). The operating temperatures for the column and injection ports were 80 and 110°C, respectively. A 100 ppm standard C_2_H_4_ gas (Airgas, Radnor Township, PA, United States) was used for calibration, and results were reported as micromoles of C_2_H_4_ per square meter of soil core surface area per hour (μmol m^–2^h^–1^).

^15^N_2_-enriched biocrust subreplicates from ^15^N_2_-enriched jars and non-enriched control biocrust subreplicates from acetylated jars were analyzed for isotopic N, and total N. Atom% ^15^N was measured using a Thermo Finnigan Delta Plus XL isotope ratio mass spectrometer with a ConFlo III preparation system at the UF/IFAS Soil and Water Sciences Elemental Analysis Laboratory, Gainesville, FL, United States. Total N was simultaneously determined using a Costech ECS 4010 CHNS-O elemental analyzer. Atom% ^15^N of headspace N_2_ was calculated by subtracting ^15^N/^14^N atom% of air from ^15^N/^14^N atom% of gas blanks. Atom% excess biomass was divided by atom% excess in headspace to calculate the fraction of N_2_-fixation derived N according to the equation adapted from Inglett (2013).


Fraction⁢of⁢N⁢derived⁢from⁢N-fixation

$$=\frac{\mathrm{atom}\%\,\mathrm{excess}\,\mathrm{in}\,\mathrm{biomass}}{\mathrm{atom}\%\,\mathrm{excess}\,\mathrm{in}\,\mathrm{headspace}}$$

$$=\frac{{\mathrm{atom}\%}_{\mathrm{Biomass}\,\mathrm{enriched}}-{\mathrm{atom}\%}_{\mathrm{Biomass}\,\mathrm{unenriched}}}{{\mathrm{atom}\%}_{\mathrm{Headspace}}-{\mathrm{atom}\%}_{\mathrm{Biomass}\,\mathrm{unenriched}}}$$

Total N in the enriched biocrust samples was then used to calculate the N_2_-fixation rate in nmols of N-N_2_ g^–1^ DW h^–1^. The conversion factor from acetylene reduction to N_2_-fixation was calculated by dividing the average biocrust acetylene reduction rate by the average enriched biocrust N_2_-fixation rate. The conversion factor was obtained separately for the summer season Grape and the fall season Citrus samples. Analogous estimates were also made using the theoretical conversion factor of 3 (Howarth et al., 1988).

### Data analysis

All data analysis was performed in R statistical software (R Core Team, 2019). First, biocrust measurements from acetylene reduction rates, microbial biomass, and nutrient measurements were compared across sites and seasons to determine the influence of interactions using general linear mixed model analysis with emmeans (Lenth, 2019) and nlme (Pinheiro et al., 2019) packages. Second, biocrusts measurements from acetylene reduction rates, microbial biomass, and nutrients were compared to bare soils within each site and season using general linear mixed model analysis with emmeans (Lenth, 2019) and nlme (Pinheiro et al., 2019) packages. A random effect for paired bare soil and biocrust samples was added to the statistical model. Biocrust and bare soil moisture, field measured light intensity, and field measured soil temperature were compared across seasons and sites using a Two-Way ANOVA with an HSD *post hoc* test. Biocrust microbial biomass, nutrients, and nutrient ratios were compared across seasons and sites using a Two-Way ANOVA with an HSD *post hoc* test. Normality was tested by the Shapiro–Wilk test for distribution and homogeneity of residuals. Non-normal data containing zeros were square root transformed, while non-normal data without zeros were log transformed. The results for general linear mixed-model analysis were reported as significant when *p* < 0.05 according to Tukey *post hoc* test. Plots were created using the ggplot2 package in R (Wickham, 2016).

To determine variables influencing N_2_-fixation rates of biocrusts, PCA was performed with the princomp function (stats 4.0.3). Each site was analyzed separately and only measurements from biocrusts were included. Prior to analysis, the data were preprocessed by filtering out zeros (Grape *n* = 21; Citrus *n* = 23), testing for multivariate normality using the mvn function from the MVN package (Korkmaz et al., 2014), and for Mardia’s multivariate skewness and kurtosis. Grape N_2_-fixation rates were log transformed, while other variables were left untransformed. For Citrus, all nutrient ratios were log transformed, moisture was root square transformed, and the rest of the variables were not transformed. Following transformations, the data were *z*-score standardized. The elbow plot and latent root criteria using base R were used to determine the number of principal components that best explained the data variation. Bootstrapped eigenvectors and loadings of at least 0.3 were used to determine the significance of loadings at the 0.01 significance level (Peres-Neto et al., 2003). PerMANOVA was performed to determine if samples were significantly separated by season using the adonis function from the vegan 2.5-7 package (Oksanen et al., 2013). Pairwise PerMANOVA was performed to determine which pairs of seasons were significantly separated from each other using wrapper function pairwise.adonis for multilevel pairwise comparison using adonis from package ‘vegan’ (Martinez Arbizu, 2020). PCA for each site was plotted with the ggbiplot function from the devtools package (currently in development by Vincent Q Vu) by only including variables with significant loadings as determined by bootstrapping. When vectors with significant loadings were not visible in the PCA plot, only one representative vector was shown.

## Results

### Qualitative biocrust characterization

**FIGURE 1:**
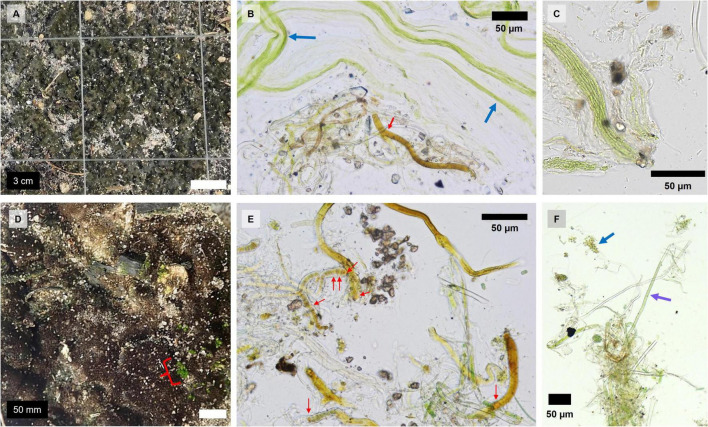
Images and micrographs of Grape and Citrus biocrusts: **(A)** Grape biocrust close-up, **(B)** filamentous algae bundles next to a heterocystous cyanobacterial filament in Grape, **(C)** non-heterocystous cyanobacterial bundles in Grape, **(D)** Citrus biocrust close-up with moss pointed out with a red bracket, **(E)** heterocystous filamentous cyanobacteria in Citrus, and **(F)** non-heterocystous cyanobacterial filament and unicellular algae in Citrus. Red arrows point to heterocystous cells, the blue arrow points to a group of unicellular algae, and the purple arrow points to a non-heterocystous cyanobacterial filament.

Biocrust visual development scale, as qualitatively determined by surface coloration and roughness (Belnap et al., 2008) was at least 5 or 6 (Figures 1A,D). Microscopic inspections identified that Grape and Citrus biocrusts were both dominated by heterocystous and non-heterocystous cyanobacteria. Grape biocrusts also had filamentous algae, whereas Citrus biocrusts also contained mosses and single-celled algae (Figures 1D,E).

### Field environmental conditions

Soils at both sites received the highest total precipitation 24 h before sampling during the summer season (Table 1), which exceeded other seasons by 2.3 mm (Citrus) and 26 mm (Grape). At the Grape site, biocrust and bare soil moisture ranged from 0.004 to 78%, with the highest average temperature in summer and the lowest average in spring, while at the Citrus site, it ranged from 0.002 to 22%, with the highest average in the winter and lowest average in fall (Table 1). At the Grape site, biocrust and bare soil moisture were significantly greater during the summer by at least 44% compared to fall, winter, and spring, even with the inclusion of added water during winter and spring collections (Table 1, *p* < 0.001). At the Grape site, biocrust and bare soil temperature ranged from 27.0 to 41.9°C, with the highest average temperature in spring and the lowest average in winter, while at the Citrus site, it ranged from 23.1 to 41.8°C, with the highest average in the summer and lowest average in winter (Table 1). At the Grape site, the light intensity on the soil surface ranged from 691 to 1741 μmol m^–2^ s^–1^ with the highest average intensity in summer and the lowest average in fall, while at the Citrus site it ranged from 504 to 1920 μmol m^–2^ s^–1^ with the highest average in summer and lowest average in fall (Table 1). Light intensity was not measured during winter at both sites.

**TABLE 1:** ANOVA results comparing nutrient averages in biocrusts between sites and seasons.

Site	Season	MBC(mg kg^–1^)	MBN(mg kg^–1^)	MBC:M BN	EC(mg kg^–1^)	EN(mg kg^–1^)	EP(mg kg^–1^)	EC:EN	EC:EP	EN:EP
Grape	Summer	1614.35 ± 656.14	102.48 ± 60.15^ab^	18.06 ± 5.52	420.93 ± 157.55	60.67 ± 32.67	156.33 ± 22.41^a^	7.69 ± 1.95^ab^	2.76 ± 1.22^c^	0.39 ± 0.22
	Fall	2562.62 ± 923.88	292.06 ± 142.80^a^	9.20 ± 1.08	511.96 ± 75.87	65.76 ± 21.64	101.38 ± 27.46^ab^	8.34 ± 2.16^ab^	5.53 ± 2.44^abc^	0.67 ± 0.22
	Winter	1433.57 ± 1175.05	121.37 ± 107.05^ab^	12.72 ± 3.03	291.83 ± 195.94	27.76 ± 19.74	105.47 ± 95.89^ab^	11.21 ± 1.89^a^	5.27 ± 5.45^abc^	0.48 ± 0.20
	Spring	1122.17 ± 1169.03	35.83 ± 61.91^b^	11.53 ± 16.29*	473.83 ± 216.34	78.17 ± 93.03	80.32 ± 14.54*b*^c^	10.72 ± 5.63^a^	5.80 ± 2.24^abc^	0.94 ± 1.12
Citrus	Summer	1578.08 ± 996.87	188.84 ± 156.33^a^	9.82 ± 2.54	173.74 ± 20.1.0	37.61 ± 11.34	48.41 ± 13.27*b*^c^	4.86 ± 1.07^b^	3.92 ± 1.48^bc^	0.84 ± 0.33
	Fall	2148.62 ± 1235.67	120.19 ± 79.96^ab^	18.19 ± 2.53	381.31 ± 366.92	43.73 ± 44.44	47.23 ± 11.62*b*^c^	9.13 ± 1.98^ab^	8.27 ± 7.54^abc^	0.96 ± 0.92
	Winter	1909.49 ± 461.38	137.38 ± 73.53^ab^	17.03 ± 7.71	285.56 ± 72.27	32.36 ± 7.53	25.90 ± 8.86^c^	8.92 ± 1.76^ab^	11.88 ± 4.23^ab^	1.43 ± 0.81
	Spring	1957.00 ± 1383.32	158.00 ± 137.57^ab^	14.49 ± 3.80	419.17 ± 123.53	58.00 ± 28.79	36.40 ± 13.67*b*^c^	8.39 ± 2.83^ab^	13.28 ± 7.67^a^	1.80 ± 1.23

Average values are followed by standard deviations.

Two-Way ANOVA was conducted with an HSD post hoc test.

*2 outliers taken out (n = 4), otherwise n = 6.

Shared letters or no letters within a column signify no statistical difference.

Significance level used: *P < 0.05.

### N_2_-fixation rates

**FIGURE 2:**
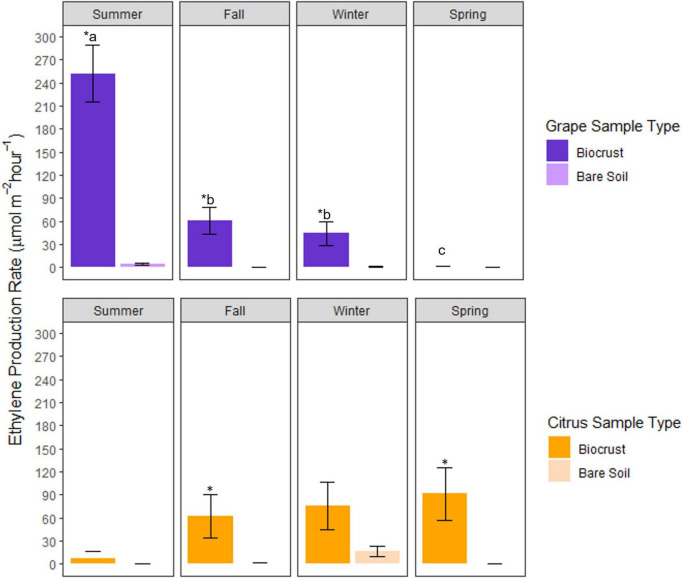
Seasonal rates of acetylene reduction (ethylene production) for biocrusts at the Grape and Citrus sites. The asterisk above the biocrust bar within a season indicates a significant difference between biocrusts and bare soils for a given sampling date, while letters indicate a significant difference between biocrusts of different seasons (*P < 0.05; *n* = 6, mean ± SE). One month prior to sampling in the summer at Citrus, controlled release fertilizer was applied at a rate of 29 kg N ha^–1^.

There were significant interactions between season, site, and sample type (biocrust or bare soil) for acetylene reduction rates (Supplementary Table 2). In Grape, average biocrust acetylene reduction rates were significantly greater in biocrusts (98%) than in bare soils during the summer, fall, and winter. In Citrus, average biocrust acetylene reduction rates were significantly greater in biocrusts (98%) during the fall, winter, and spring (Figure 2).

Grape biocrusts had higher average acetylene reduction rates than Citrus biocrusts. Grape biocrust acetylene reduction rates ranged from undetectable to 401 μmol m^–2^ h^–1^ with the highest average rate in summer (260 μmol m^–2^ h^–1^) and the lowest average rate in spring (4.19 μmol m^–2^ h^–1^) (Figure 2). Acetylene reduction rates of Citrus biocrusts ranged from undetectable to 326 μmol m^–2^ h^–1^ with the highest rate in winter (78 μmol m^–2^ h^–1^) and the lowest in summer (13 μmol m^–2^ h^–1^).

Average Grape biocrust acetylene reduction rates decreased from summer to spring, while average Citrus biocrust acetylene reduction rates increased from summer to spring. Grape biocrust acetylene reduction rates were significantly greater during the summer when compared to Grape biocrust acetylene reduction rates during fall, winter, and spring (Figure 2). While not significant, there was a trend of increasing acetylene reduction rates from summer to spring in Citrus (Figure 2).

The C_2_H_4_:N_2_ conversion factor was lower in Citrus (1.22 ± 0.23, standard deviation) than in Grape (2.69 ± 0.92), leading to higher average annual N input estimates from N_2_-fixation in Citrus. Estimates of biocrust N_2_-fixation rates using both the experimental and theoretical 3:1 ratios are shown in Table 2. Assuming a constant C_2_H_4_:N_2_ conversion factor and using experimental ratios, the N_2_-fixation rates in Grape ranged from 1.87 × 10^–6^ to 4.18 × 10^–3^ g N m^–2^ h^–1^, while in Citrus it ranged from 2.05 × 10^–6^ to 7.49 × 10^–3^ g N m^–2^ h^–1^. Based on the assumption that phototrophic diazotrophs were engaged in N_2_-fixation for 10 light hours, we estimated the daily N_2_-fixation rate of these agroecosystem biocrusts to be 6–32 mg of N per day. Averaging N_2_-fixation inputs separately for each season and site, and then adding for an annual estimate with the assumption that biocrust agroecosystems had 12.5% biocrust soil surface area coverage, Citrus biocrusts were estimated to provide 8.1 kg N ha^–1^ year^–1^, while Grape could provide 4.9 kg N ha^–1^ year^–1^.

**TABLE 2:** N_2_-fixation rate comparison of this study’s rates to literature rates.

Type	Incubation	Ethylene production rate (μmols C_2_H_4_ m^–2^h^–1^)	N_2_-Fixed (mg N m^–2^h^–1^), Conversion ratio*	Source
		
		Average or Range		
Cyanobacterial dominated, southwestern USA deserts, variable soil	Lab, 4–8 h at 20°C in light	10–100	0.6–11, 1 *0.2-4, 3*	Strauss et al., 2012
Cyanobacterial dominated, Oman desert, loamy sand and silty loam	Lab, 10 h at 35°C in light and dark	58.5	*1.1, 3*	Abed et al., 2010
All succession types with high deposition (16–25 kg N ha^–1^ year^–1^) and average rainfall (730–970 mm year^–1^), sandy	Lab, 4 h at 26 °C in light	95–115	*1.7–2.0, 3*	Veluci et al., 2006
Single lichen species dominated, MOAB, UTAH	Lab, 4 h, unknown Temperature, in light	4–16	*0.07–0.30, 3*	Torres-Cruz et al., 2018
Canada grassland dark biocrusts	Lab, 4-6 h, 21 °C	116–173	108–162, 0.06 *2.2–3.2, 3*	Caputa et al., 2013
Dark biocrust from young grape field in Citra, FL, United States	Field, 2 h, 29–39°C in light	58–260	0.6–2.7, 2.7 *0.5–2.4, 3*	This study: Grape**
Dark biocrust from young citrus field in Lake Alfred, FL, United States	Field, 2 h, 24–35°C in light	74–137	1.7–3.1, 1.2 *0.7–1.3, 3*	This study: Citrus**

*Calculated using either a provided experimental moles of ethylene produced to moles of N_2_ fixed ratio or a ratio chosen from literature by the authors.

**Only average rates that were significantly higher than bare soils were included (Figure 2).

N inputs calculated using theoretical conversion ratio are italicized.

### Microbial biomass nutrients and ratios

Microbial and extractable carbon was significantly greater in Grape biocrusts compared to bare soil only during the fall, while at the Citrus, MBC was significantly greater in biocrusts compared to bare soil during the fall, winter, and spring seasons (Supplementary Figure 3). Grape biocrust MBC ranged from 19 to 4036 mg kg^–1^ with the highest concentration in fall (2563 mg kg^–1^) and lowest in spring (1122 mg kg^–1^). Citrus biocrust MBC ranged from 232 to 3998 mg kg^–1^ with the highest concentration in fall (2149 mg kg^–1^) and lowest in summer (1578 mg kg^–1^) (Supplementary Figure 3).

Grape MBN was significantly greater in biocrusts compared to bare soil in the fall and winter, while MBN was not significantly different between Citrus biocrusts and bare soils in all seasons (Supplementary Figure 3). Grape biocrust MBN ranged from 2 to 520 mg kg^–1^ with the highest concentration in winter (121 mg kg^–1^) and lowest in spring (36 mg kg^–1^). Citrus biocrust MBN ranged from 16 to 480 mg kg^–1^ with the highest concentration in summer (189 mg kg^–1^) and lowest in fall (120 mg kg^–1^). There was also a significant interaction between site and season for biocrust MBN (Supplementary Table 1), where Grape biocrust MBN was significantly greater in the fall compared to the spring (Table 1).

Grape biocrust MBP ranged from undetectable to 83 mg kg^–1^ with the highest concentration in summer (29 mg kg^–1^) and lowest in winter (10 mg kg^–1^). Citrus biocrust MBP ranged from undetectable to 68 mg kg^–1^ with the highest concentration in spring (29.5 mg kg^–1^) and lowest in winter (3 mg kg^–1^) (Supplementary Figure 3). Due to the high variability and MBP being below the detection limit in 30% of samples, it was not analyzed for significant differences.

There were different seasonal patterns in MBC:MBN ratios in Grape and Citrus. Mass-based MBN:MBP and MBC:MBP ratios were excluded because 30% of MBP values were below the detection limit. Grape biocrust MBC:MBN ranged from 6 to 44, while Citrus biocrust MBC:MBN ranged from 7 to 30. The highest biocrust MBC:MBN in Grape was during the summer (18.1 ± 5.52), while in Citrus it was during the fall (18.2 ± 2.53). The lowest biocrust MBC:MBN in Grape was in fall (9.2 ± 1.08), while in Citrus it was during the summer (9.82 ± 2.54) (Table 1).

### Extractable nutrient concentrations and ratios

In general, EC, EN, and EP were higher in biocrusts than in bare soils in Grape and Citrus. EC was significantly greater in biocrusts than in bare soils during the summer (Grape), fall (Grape and Citrus), and spring (Citrus) (Supplementary Figure 4). Grape biocrust EC ranged from 106 to 780 mg kg^–1^ with a high in fall (512 mg kg^–1^) and a low in spring (292 mg kg^–1^). Citrus biocrust EC ranged from 83 to 1107 mg kg^–1^ with a high in spring (419 mg kg^–1^) and a low in summer (174 mg kg^–1^).

EN was significantly greater in biocrusts than in bare soils during the summer (Citrus), fall (Grape), winter (Citrus), and spring (Citrus). Grape biocrust EN ranged from 7 to 255 mg kg^–1^ with a high in spring (78 mg kg^–1^) and a low in winter (28 mg kg^–1^). Citrus biocrust EN ranged from 9 to 132 mg kg^–1^ with a high in spring (58 mg kg^–1^) and a low in winter (32 mg kg^–1^).

There was a significant interaction between site and sample type for EP (Supplementary Table 2). EP tended to be higher in Grape biocrusts than in Citrus biocrusts. EP was significantly greater in biocrusts than in bare soils during the summer (Grape), fall (Citrus), and winter (Grape). EP in Grape biocrusts decreased from summer to spring, while in Citrus there was no significant seasonal trend (Table 1). Grape biocrust EP ranged from 36 to 268 mg kg^–1^ with a high in summer (156 mg kg^–1^) and a low in spring (80 mg kg^–1^). Citrus biocrust EP ranged from 12 to 67 mg kg^–1^ with a high in summer (48 mg kg^–1^) and a low in winter (26 mg kg^–1^).

No strong seasonal patterns or significant differences between sites, biocrusts and bare soils, or seasons were detected in extractable nutrient ratios. Due to undetected EN and EP, certain ratios were excluded from the following comparisons. Grape biocrust EC:EN ranged from 3 to 18, while Grape bare soil EC:EN ranged from 7 to 15. Citrus biocrust EC:EN ranged from 3 to 13, while Citrus bare soil EC:EN ranged from 1 to 10. Grape biocrust EN:EP ranged from 0.05 to 3, while Grape bare soil EN:EP ranged from 0.2 to 0.6. Citrus biocrust EN:EP ranged from 0.2 to 4, while Citrus bare soil EN:EP ranged from 0.2 to 2. Highest average Grape biocrust EC:EN was in winter (11.2 ± 1.89), while the lowest Grape biocrust EC:EN was in summer (4.9 ± 1.07). Highest average Citrus biocrust EC:EN was in fall (9.1 ± 1.98), while the lowest Citrus biocrust EC:EN was in summer (4.86 ± 1.07). Highest average Grape biocrust EN:EP was in spring (0.9 ± 1.12), while the lowest Grape biocrust EN:EP was in summer (0.4 ± 0.22). Highest average Citrus biocrust EN:EP was in spring (1.8 ± 1.23), while the lowest Citrus biocrust EN:EP was in summer (0.8 ± 0.33) (Table 1).

### Relationship between N_2_-fixation activity, nutrients, and environmental conditions

Variation in actively N-fixing biocrusts in Grape and Citrus was explained by the variation in environmental conditions, nutrients, biomass, and acetylene reduction rates. Biotic variables (MBC, MBN, MBC:MBN), nutrient variables (EC, EN, EP, EC:EN, EN:EP, EC:EP), acetylene reduction rates, and environmental variables (soil moisture, soil temperature) measured in N_2_-fixing biocrusts were included in the PCA. At Grape, MBC:MBN, extractable nutrients, extractable nutrient ratios, and soil moisture were significant drivers of variation, as indicated by the significance of loadings (Supplementary Table 3). Based on significant loading, PC1 in Grape may represent nutrients, PC2 may represent N_2_-fixation activity, and PC3 may represent microbial biomass. At Citrus, microbial biomass, MBC:MBN, EC, EN, EC:EP, EN:EP EC:EN, and temperature were significant drivers of variation (Supplementary Table 2). Based on significant loading, Citrus PC1 may represent nutrients and microbial biomass carbon, PC2 may represent N_2_-fixation activity, and PC3 may represent microbial biomass nitrogen, nutrients, and environmental variables.

Actively N_2_-fixing biocrust samples in Grape (46%) and Citrus (29%) were significantly separated based on the season (Supplementary Table 4). N_2_-fixing biocrust samples in summer significantly differed from the fall and winter seasons in both Grape and Citrus (Supplementary Table 4). Grape summer samples were significantly different from fall and winter samples mainly because of higher EC, EN, MBC:MBN, EN:EP, and EC:EP but lower EC:EN (PC1); and higher acetylene reduction rates, soil moisture, and EP, but lower EC:EN (PC2, Supplementary Table 3). Citrus summer samples were significantly different from fall and winter samples mainly because of lower MBC, EC, EN, EN:EP, and EC:EP (PC1); and lower acetylene reduction rates, MBC:MBN, and EC:EN but higher soil temperatures (PC2, Supplementary Table 3).

## Discussion

Agroecosystem biocrusts were identified as dark algal biocrusts in Grape and dark cyanobacterial biocrusts in Citrus. During select seasons at both sites, biocrust N_2_-fixation rates, MBC, MBN, EC, EN, and EP were significantly higher than in bare soils. The N_2_-fixation rates in Grape significantly decreased from summer to spring, while in Citrus an opposite but non-significant trend was measured. In addition, in Grape biocrust MBN increased from summer to fall, but then decreased from fall to spring. The N_2_-fixing biocrust acetylene reduction rates, nutrient concentrations, microbial biomass, soil moisture, and soil temperature significantly differed across seasons. Higher soil moisture and higher EP were associated with higher N_2_-fixation rates in Grape, while lower soil temperatures and higher EC:EN ratios were associated with higher N_2_-fixation rates in citrus biocrusts.

The Florida agroecosystem biocrusts in this study appeared to be dark algal and cyanobacterial biocrusts based on assessments using microscopy and the visual biocrust development scale (Belnap et al., 2008). In addition, despite large climatic differences, subtropical Florida agroecosystem biocrusts had average MBC values similar to natural cyanobacterial biocrusts of a semi-arid Mediterranean desert (Miralles et al., 2012). The lower range of MBN from these agroecosystem biocrusts was also similar to the MBN of natural lichen-dominated biocrusts of Mediterranean grassland (Castillo-Monroy et al., 2010).

Biocrusts in both citrus and grape agroecosystems fixed atmospheric N_2_, with rates within the ranges recorded in both arid and mesic natural ecosystem biocrusts (Table 2), including biocrusts in restored Florida wetlands (Liao and Inglett, 2014). To calculate rates of N_2_-fixation, we converted ethylene production rates into mg of N inputs by using an experimental ratio for moles of C_2_H_4_ to moles of N_2_. Grape biocrusts fixed between 0.6 and 2.7 mg N m^–2^ h^–1^, while Citrus biocrusts fixed between 1.7 and 3.1 mg N m^–2^ h^–1^ (Table 2). In citrus and grape agroecosystems, biocrust could potentially contribute between 0.6 and 3 mg of N m^2^ h^–1^ through N_2_-fixation based on experimental conversion ratios (Table 2). As these agroecosystem biocrusts appear to be similar to dark cyanobacterial biocrusts, cyanobacteria could be the dominant contributors to N_2_-fixation of these agroecosystem biocrusts. Based on the assumption that phototrophic diazotrophs were engaged in N_2_-fixation for 10 light hours, we estimated the daily N_2_-fixation rate of these agroecosystem biocrusts to be 6–32 mg of N per day, which constitutes less than 1% of fertigation for a single citrus tree from our site per application (4–5 g N, based on fertigation information specific to citrus) (Obreza and Morgan, 2020). It should be noted, however, that this is likely an underestimate as these rates are single time point measurements in a diel cycle, and therefore do not include potential dark or nighttime N_2_-fixation.

If it is assumed that biocrust agroecosystems had 100% coverage over a hectare, Citrus biocrusts could contribute 64 kg N ha^–1^ year^–1^, while Grape biocrusts could provide 39 kg N ha^–1^ year^–1^ per hectare of biocrust. However, while in natural ecosystems up to 70% of soil surface can be covered with biocrusts (Ferrenberg et al., 2015), agroecosystem biocrusts tend to grow in crop interspaces and only between crop rows, leaving at most 12.5% of the total field area available for biocrust growth (based on field observations). Assuming 12.5% biocrust soil surface area coverage, Citrus biocrusts are estimated to provide 8.1 kg N ha^–1^ year^–1^, while Grape could provide 4.9 kg N ha^–1^ year^–1^, which satisfies 7 and 14% of total yearly N input, respectively. Regardless of potential overestimation and underestimation due to lack of night and diurnal measurements, these estimated rates fit well within the expected N input contribution of natural ecosystem biocrusts (Malam Issa et al., 2001; Belnap, 2002; Billings et al., 2003; Russow et al., 2005; Housman et al., 2006; Holst et al., 2009).

**FIGURE 3:**
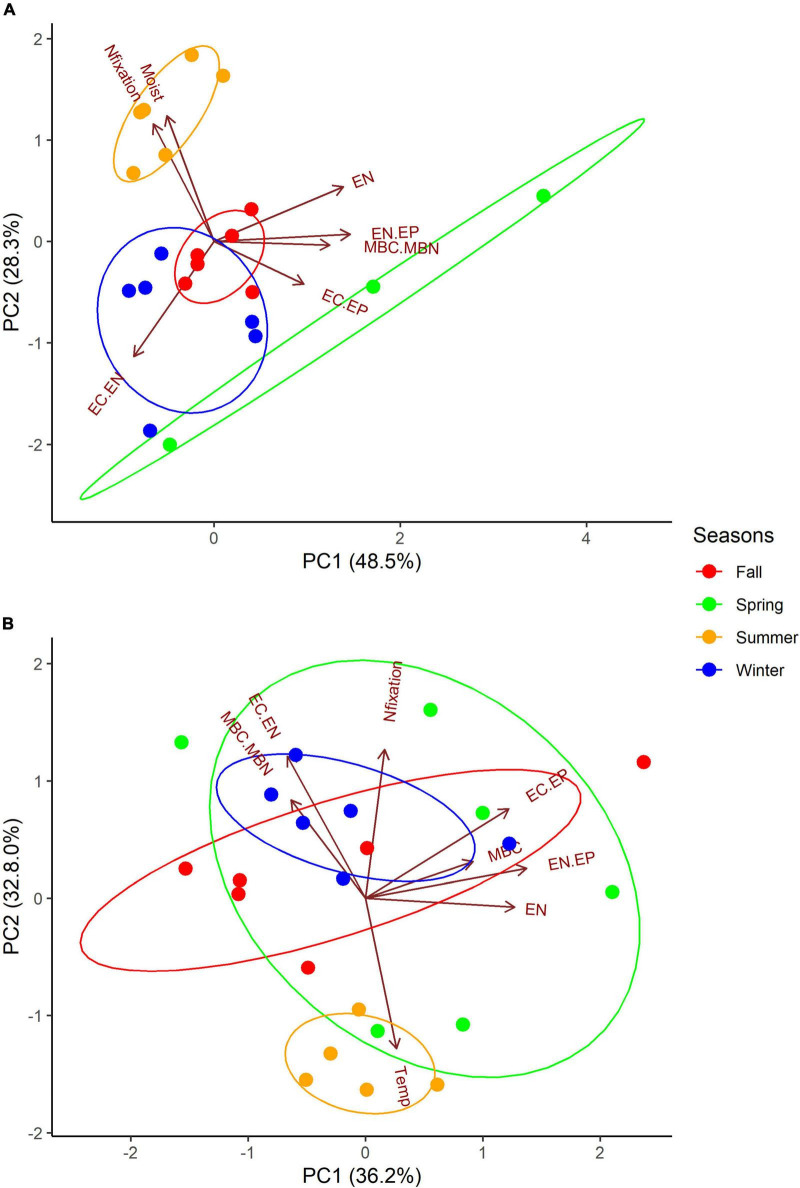
Principle component analysis (PCA) of seasonally N_2_-fixing biocrusts from **(A)** Grape (*n* = 21) and **(B)** Citrus (*n* = 23). Vectors represent variables with significant loadings used to generate Euclidean distance: N_2_-fixation (Nfixation), biocrust moisture (Moist), soil temperature (Temp), extractable nutrients (EC, EN, EP, EC.EN) and their ratios (EN.EP, EC.EP), and microbial nutrients (MBC, MBN) and their ratio (MBC.MBN). In Grape, the following vectors represent multiple variables: Nfixation (EP) and EN (EC). In Citrus, the following vector represents two variables: EN (EC). Ellipses are drawn around seasons at 98% probability. Vector loadings and their significance with all variables included are shown in Supplementary Table 3. PerMANOVA results of differences based on the season are shown in Supplementary Table 4.

Higher precipitation favors higher N_2_-fixation rates in natural ecosystem biocrusts, and if coupled with higher moisture, warmer temperatures can also lead to higher N_2_-fixation rates (Zhao et al., 2010; Caputa et al., 2013). Similar to dark and light cyanobacterial biocrusts from Southwestern U.S. deserts (Belnap, 2002), N_2_-fixation activity in Grape biocrusts peaked during the season of higher precipitation and after a high precipitation event (Figure 2 and Table 3). In the summer, 46% of Grape N_2_-fixation rates were significantly higher compared to fall and winter seasons potentially because of higher moisture. Biocrust moisture was also significantly higher in the summer and was one of the significant drivers of variation along PC2 of Grape N_2_-fixing biocrust samples (Figure 3 and Supplementary Tables 3, 4).

**TABLE 3:** Environmental parameters of field incubations.

Site	Collection date	Mean soil temp. during incubation (°C)	Mean light intensity on soil surface (μmol m^–2^ s^–1^)	Biocrust and bare soil moisture (%)	Total rainfall (24 h within sampling time) (mm)*
Grape	Summer	36.08± 1.38*^b^*	1551.02± 119.12*^a^*	47.17± 16.80*^a^*	26
	Fall	33.55 ± 0.94*^c^*	876.84± 84.49*^d^*	2.49± 2.01*^b^*	0.5
	Winter	29.39± 1.75*^d^*	NA	1.72± 1.95*^b#^*	0.0
	Spring	38.76± 1.98*^a^*	1402.50 ± 47.30*^ab^*	0.87± 0.004*^b#^*	0.76
Citrus	Summer	35.31 ± 1.70*^bc^*	1564.53± 286.50*^a^*	2.61± 1.94*^b^*	2.3
	Fall	30.88± 2.32*^d^*	1083.5± 166.29*^cd^*	0.74± 0.63*^b^*	0.0
	Winter	24.45± 1.05*^e^*	NA	2.89± 5.93*^b^*	0.0
	Spring	33.98 ± 5.06*^c^*	1291.92± 467.70*^bc^*	1.40± 0.02*^b^*	0.0

Average values are followed by standard deviations.

Letters represent significant differences according to two-way ANOVA with an HSD post hoc test.

NA, measurements were not taken.

*FAWN – This data was retrieved from Florida Automated Weather Network.

^#^Deionized water was added until visual saturation during this time point to the bare soil and biocrusts before core collection.

Irrigation management may also be responsible for the seasonal pattern differences in microbial biomass and N_2_-fixation rates between Grape and Citrus biocrusts. For example, rainfall may have impacted N_2_-fixation rates more for the drip-irrigated Grape system (Table 3 and Figure 2), while Citrus was irrigated with a higher-intensity microjet system. Thus, there was likely less variability of surface soil moisture at Citrus, and a greater dependence on precipitation at Grape, potentially explaining the lower Grape biocrust N_2_-fixation rates in drier seasons of fall, winter, and spring (Figures 2, 3) and the weak relationship between soil moisture and N_2_-fixation rates (Figure 3) at Citrus.

Despite regular N fertilizer inputs, biocrusts in these perennial agroecosystems had detectable N_2_-fixation activity for most of the year (Figure 2). While the presence of available N can inhibit N_2_-fixation (Nordlund and Ludden, 2004; Masepohl and Forchhammer, 2007), the continued N_2_-fixation by these agroecosystem biocrusts indicates that these organisms may tolerate a threshold of N fertilizer in their environment. For example, below the threshold of 55 lbs acre^–1^, N_2_-fixation of lab-grown cyanobacteria sourced from agroecosystem biocrust continued to fix N_2_ at a steady rate (Peng and Bruns, 2019). In Florida citrus, this threshold of 55 lbs acre^–1^ (Peng and Bruns, 2019) would be equivalent to the lowest recommended fertilizer rate for a tree that has been in the orchard for 1–3 years (Obreza and Morgan, 2020), and the trees in our study were less than 3 years old during our sample collections. However, controlled release fertilizer application may have reduced N_2_-fixation rates of Citrus biocrusts in the summer (lowest N_2_-fixation rates and highest MBN) which occurred 1 month after application of a controlled release fertilizer containing ammonium and nitrate (Supplementary Table 1). This could mean that within a month of controlled release fertilizer application, the microbial community assimilated the fertilizer N and did not require biological N_2_-fixation to meet N demands. Two months later, however, Citrus had significantly higher N_2_-fixation rates and MBN dropped by approximately 60 mg kg^–1^ (Table 1), suggesting an ability and necessity to resume N_2_-fixation activity. While Citrus received weekly fertigation, the controlled release fertilizer form may have a stronger negative effect on biocrust N_2_-fixation than the liquid form due to its higher concentration of N (Supplementary Table 1). The exponential increase in released N (Sempeho et al., 2014) might have resulted in a longer period of higher N concentrations in proximity to biocrusts. In contrast, liquid fertilizer N, especially the nitrate form applied to sandy soils in this Citrus (Gaines and Gaines, 1994), may leach quickly (Kadyampakeni et al., 2018), reducing the concentration of N and allowing for higher N_2_-fixation activity.

Grape and citrus have different fertilizer requirements, which may have also resulted in different N_2_-fixation rates between the sites (Figure 3). For example, the vineyards in this study received a 1:1 ratio of N to phosphate, versus 7:2 for citrus (Supplementary Table 1). In the Grape biocrusts, as EP increased, N_2_-fixation also tended to increase, possibly suggesting that higher EP led to higher N_2_-fixation rates (Figure 3). Phosphorus addition is known to stimulate N_2_-fixation activity in highly weathered soils such as Oxisols and Ultisols (Reed et al., 2011), and increased P inputs can shift an ecosystem from P to N limitation, resulting in increased N_2_-fixation activity of cyanobacterial communities and biocrusts (Inglett et al., 2009; Liao and Inglett, 2012). Oxisols and Ultisols tend to be more common in tropical climates, and while our sites are located in the subtropics, they have Entisols that are not known to commonly be P limited. However, the biocrusts on a smaller scale could still be P limited due to having different properties than the soil below.

Extractable nutrients and their ratios explained 33–38% of the total variation in N_2_-fixing biocrusts (Figure 3), which is not surprising because nutrient status has been shown to govern N_2_-fixation activity (Inglett et al., 2004, 2009, 2011). The N:P of the Grape fertilizer was lower than at Citrus (Supplementary Table 1), so fertilization likely led to the N limitation in Grape. A higher EC:EN was also associated with higher N_2_-fixation rates in Citrus (Figure 3 and Supplementary Table 3), and higher EC and EC:EN ratios coincided with seasons of higher N_2_-fixation activity in Citrus (fall, winter, and spring) (Table 2). Therefore, Citrus biocrusts might be experiencing C and N colimitation that regulated N_2_-fixation. Despite the fertilizer differences between Grape and Citrus, EN:EP ratio of biocrusts at both sites did not exceed 4, which was also true for a desert biocrust N:P (Zhou et al., 2016). Such a ratio is well below the threshold for N limitation of grassland soil microorganisms (Griffiths et al., 2012), suggesting that the biocrusts might have been experiencing N limitation at both sites.

Differences in environmental conditions, planting history, and fertilization management likely result in organism level differences between Citrus and Grape biocrusts. Diazotrophic community composition may be responsible for the seasonal pattern differences in biomass and N_2_-fixation rates between Grape and Citrus biocrusts. Grape and citrus have different disturbance histories from planting, which could explain the differences in their biocrust morphologies. Using the qualitative indicators of potentially different organism compositions in biocrusts (Belnap et al., 2008), Grape biocrusts had a homogeneous dark green color after wetting, while Citrus biocrusts had heterogeneous dark green and dark brown coloration after wetting (Figure 1). Certain organisms were also not detected microscopically in both sites; for example, filamentous algae were specific to the Grape biocrusts, while single-celled algae and mosses were specific to Citrus biocrusts. The older tree age of Citrus (i.e., more time since major soil disturbance) compared to grape (2 years > 2 months) might have allowed Citrus biocrusts to become more homogeneous in coloration and to contain mosses. Also, despite similar climatic conditions, N_2_-fixation rates followed contrasting seasonal patterns in Grape and Citrus (Figure 2), indicating potentially different organism communities and growth/senescence cycles which may be supported by MBN seasonal patterns (Supplementary Figure 3). Finally, the conversion factor for moles of C_2_H_4_ to moles of N_2_ was not the same for the two sites (3 for Grape and 1 for Citrus), suggesting that different communities of organisms were involved in N_2_-fixation at each site. Due to the variety of diazotroph nitrogenases, the conversion factor can differ between not only phyla but also species of cyanobacteria, as it did between *Anabaena* culture (4–5) and *Nostoc* culture (0.1–0.5) (Liengen, 1999). Additionally, conversion factors lower than 2 can be the result of N_2_-fixers with alternative nitrogenases that use vanadium or iron cofactors, which is common for asymbiotic soil N_2_-fixers (Bellenger et al., 2014). Therefore, based on the experimentally derived conversion ratios, diazotrophs from Grape and Citrus could contain organisms that use different nitrogenases.

## Conclusion

Despite regular N fertilizer inputs, biocrusts in Florida citrus and grape agroecosystems maintained N_2_-fixation activity within ranges of natural biocrusts. These agroecosystem biocrusts have the potential to supplement available N for the crops through N_2_-fixation activity. While biocrusts could contribute less than 1% of the daily fertigation requirement of 1–3-year-old citrus, their continued N_2_-fixation activity during favorable conditions could contribute 7-14% of yearly N requirements for perennial crops such as grape and citrus.

To arrive at more accurate N_2_-fixation rates and patterns for better estimates of N inputs from N_2_-fixation on a crop field yearly scale, studies across more time and spatial scales are necessary. Additional diel N_2_-fixation rate measurements would show if there are contributions from non-phototrophic diazotrophs (dark), or more N_2_-fixation activity during other times of the day that this study did not consider. This study used 3 cm diameter cores for N_2_-fixation rate measurements, but there is a need to scale up to the whole field area for more accurate N input estimates, which could be done with more and larger core collections, biocrust percent cover measurements, and potentially paired with remote sensing technology as was done for biocrusts in natural ecosystems (Havrilla et al., 2020).

As hypothesized, crop-specific fertilization and irrigation management appeared to impact N_2_-fixation rates as fertilization and soil temperatures were the main controls of N_2_-fixation in citrus systems, while P and soil moisture were the main controls in vineyards. The differences in crop management and patterns of microbial biomass and N_2_-fixation patterns point to the possibility that the microbial communities of these crops could be distinct. To further explore microbial community differences between grape and citrus biocrusts, biocrust DNA could be analyzed for community composition and diversity.

Detailed analysis of biocrust microbial communities could help identify agroecosystem biocrusts N_2_-fixing organisms and their N_2_-fixation strategies, which could be important for developing management strategies to encourage N_2_-fixation activity in biocrusts. To further examine the relationship between N and P fertilization and N_2_-fixation in agricultural biocrusts, experiments with controlled N and P addition would better establish the potential for nutrient thresholds for suppression (N) and stimulation (P) of biocrust N_2_-fixation rates. Ultimately, further agroecosystem biocrust studies should aid in determining whether biocrust N inputs make any substantial improvements to crop productivity, or at least help satisfy N crop requirements more sustainably.

## Data availability statement

The raw data supporting the conclusions of this article will be made available by the authors, without undue reservation.

## Author contributions

PI, SS, and KS designed the experiment. KS conducted the experiment, processed the samples, analyzed the data, and wrote the manuscript. SS and PI revised the manuscript. All authors contributed to the article and approved the submitted version.

## Footnotes

1
https://fawn.ifas.ufl.edu/data/
