# Supplementary material for: Contrasting seasonal patterns and factors regulating biocrust N2-fixation in two Florida agroecosystems
Source: Front Microbiol. 2022 Aug 3;13:892266. doi: 10.3389/fmicb.2022.892266 (PMC9381872; doi:10.3389/fmicb.2022.892266)
Supplement: Supplementary file 1 [file Data_Sheet_1.docx]

**Supplementary Figure 1.**

**Sampling and analysis schematic.**


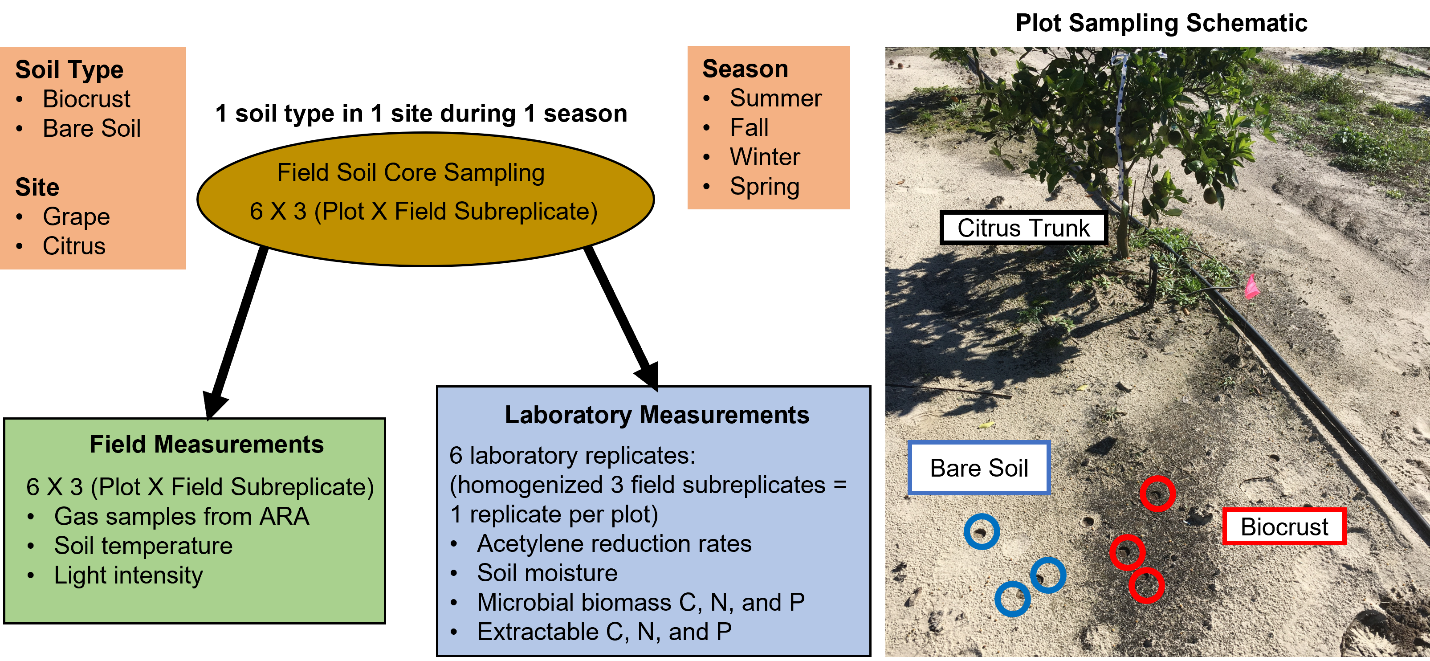


**Supplementary Figure 2.**

Acetylene reduction assay jar set-up


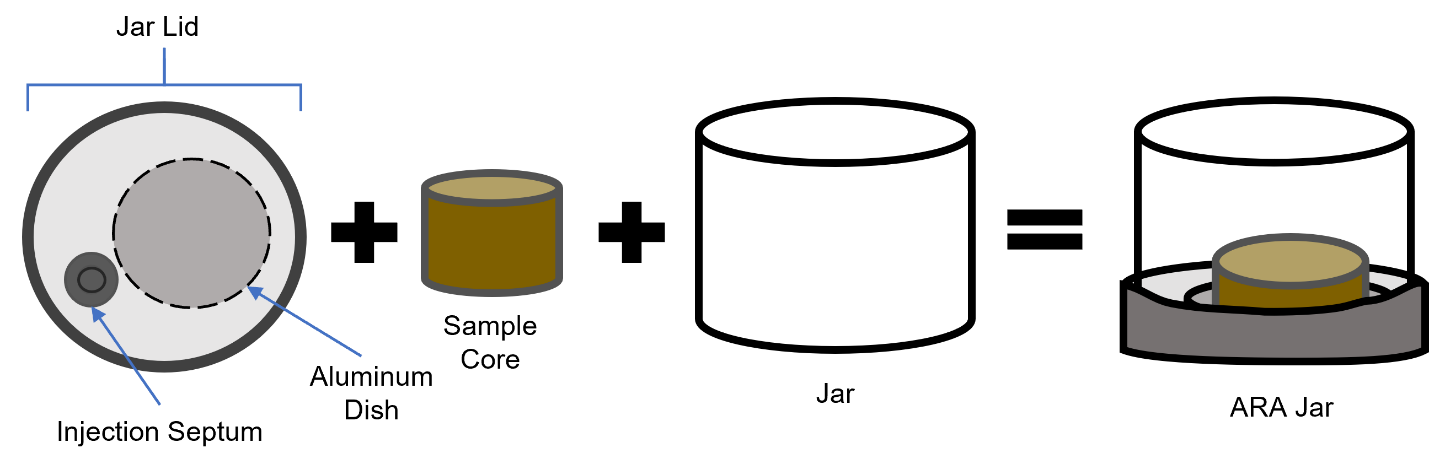


**Supplementary Figure 3.**

Seasonal microbial biomass (a) N, (b) C, and (c) P for biocrusts at the Grape and Citrus sites. Asterisk above biocrust bar within a season indicates significant difference between biocrusts and bare soils for a given sampling date (p < 0.05; n = 6, mean ± SE).

**
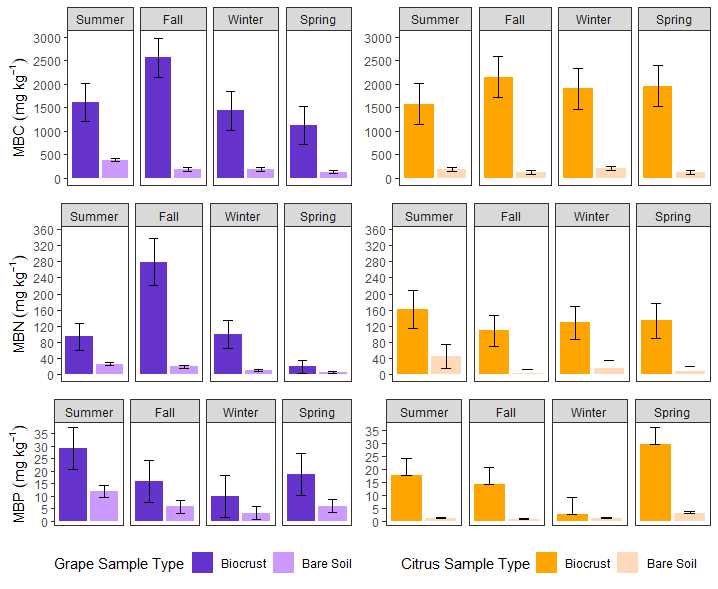
**

*

*

*

*

*

*

**Supplementary Figure 4.**

Seasonal extractable (a) C, (b) N, and (c) P at the Grape and Citrus sites. Asterisk above biocrust bar within a season indicates significant difference between biocrusts and bare soils for a given sampling date (p < 0.05; n = 6, mean ± SE).


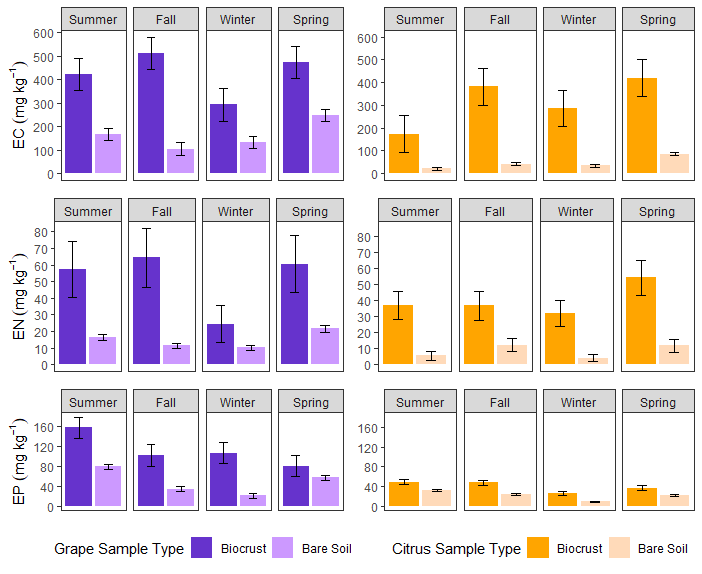


*

*

*

*

*

*

*

*

*

*

*

**Supplementary Table 1.** Summary of fertilizer inputs for Grape and Citrus sites

| Site | Fertilizer Formula* | Application Dates | N Fertilizer Rate per Application  (kg N ha^-1^) | P Fertilizer Rate per Application  (kg P ha^-1^) | (Season) Days Collected Post Application |
| --- | --- | --- | --- | --- | --- |
| Grape | Dry granules: 10-10-10* | Jun 9, 2019 | 11.2 | 4.88 | (Summer) 70 |
|  |  | - | - | - | (Fall) 141 |
|  |  | - | - | - | (Winter) 217 |
|  |  | March 10, 2020 | 11.2 | 4.88 | (Spring) 71 |
| Citrus | Liquid fertigation: 5-0-7, 7-2-7 | Once weekly, alternating formula | 2.40, 2.88 | 0, 0.359 | (Summer) 3 |
|  |  |  |  |  | (Fall) 4 |
|  |  |  |  |  | (Winter) 17 |
|  |  |  |  |  | (Spring) 2 |
|  | Slow release: 12-4-8 | July 2019 | 29.2 | 4.24 | (Summer) at least 30 |
|  |  | - | - | - | (Fall) at least 90 |
|  |  | - | - | - | (Winter) |
|  |  | - | - | - | (Spring) |

The dash (-) signifies no application.

* N-PO_4_^3-^-K_2_O

**Supplementary Table 2.** Summary of generalized linear mixed model results

|  | N-fixation | MBC | MBN | EC | EN | EP |
| --- | --- | --- | --- | --- | --- | --- |
| Time:Type | ***** | NS | NS | NS | NS | NS |
| Site:Time | ******* | NS | ****** | NS | NS | NS |
| Site:Type | ******* | NS | NS | NS | NS | ***** |
| Time:Type:Site | ******* | NS | NS | NS | NS | NS |
| Site | ********* | NS | NS | NS | NS | ********* |
| Type | NS | ****** | ******* | ******* | ****** | NS |
| Time | ******** | NS | NS | NS | NS | NS |

NS no significant difference

* P < 0.05; ** P < 0.01, *** P < 0.001

**Supplementary Table 3.** Grape and Citrus loadings of first 3 PCs, variation explained, and their significance

|  | Grape | | | Citrus | | |
| --- | --- | --- | --- | --- | --- | --- |
|  | PC1 | PC2 | PC3 | PC1 | PC2 | PC3 |
| *Variation Explained* | 38.1% | 23.6% | 17.2% | 32.5% | 25.0% | 16.4% |
| MBC^2^ | 0.282 | 0.255 | **0.432** | **0.357*** | 0.067 | 0.216 |
| MBN | 0.037 | 0.241 | **0.584** | 0.290 | 0.269 | **0.348** |
| EC | **0.418*** | 0.072 | 0.011 | **0.396** | -0.094 | **-0.314** |
| EN^1^ | **0.434*** | 0.099 | -0.122 | **0.397** | 0.143 | **-0.325** |
| EP | -0.115 | **0.453** | -0.074 | -0.178 | 0.249 | -0.362 |
| MBC:MBN^1,2^ | **0.332** | -0.124 | -0.286 | -0.136 | **-0.401** | -0.270 |
| EC:EN^1,2^ | **-0.314** | **-0.394** | 0.021 | -0.093 | **-0.499** | -0.048 |
| EN:EP^1,2^ | **0.435** | -0.108 | -0.020 | **0.433** | -0.006 | -0.083 |
| EC:EP^1,2^ | **0.304** | -0.261 | 0.202 | **0.440** | -0.192 | -0.068 |
| Moisture^1^ | -0.090 | **0.418** | **-0.353** | -0.007 | 0.130 | **0.519** |
| Temperature^2^ | 0.172 | 0.181 | **-0.439** | -0.040 | **0.452** | **-0.347** |
| N-fixation^1,2^ | -0.115 | **0.446** | 0.099 | 0.179 | **-0.405** | 0.143 |

**Bolded** – Bootstrapping significant at 0.1 p-level

**Bolded*** – Bootstrapping significant at 0.05 p-level

^1^ Chosen variable for Grape PCA plot

^2^ Chosen variable for Citrus PCA plot

**Supplementary Table 4.** Grape and Citrus perMANOVA and Pairwise perMANOVA results based on seasons

|  | Grape | | | | Citrus | | | |
| --- | --- | --- | --- | --- | --- | --- | --- | --- |
|  | **F Statistic** | **R^2^** | **P Value** | **Bonferoni Adjusted P-value** | **F Statistic** | **R^2^** | **P Value** | **Bonferoni Adjusted P-value** |
| Global Seasons | 4.908 | 0.464 | 0.001 | NA | 2.555 | 0.287 | 0.001 | NA |
| Summer vs Fall | 9.872 | 0.497 | 0.003 | ***0.018*** | 3.052 | 0.253 | 0.001 | ***0.006*** |
| Summer vs Winter | 6.312 | 0.387 | 0.001 | ***0.006*** | 6.914 | 0.434 | 0.001 | ***0.006*** |
| Summer vs Spring | 6.037 | 0.463 | 0.013 | 0.078 | 3.475 | 0.279 | 0.013 | 0.078 |
| Fall vs Winter | 3.074 | 0.235 | 0.046 | 0.276 | 1.574 | 0.136 | 0.146 | 0.876 |
| Fall vs Spring | 4.028 | 0.365 | 0.011 | 0.066 | 0.971 | 0.089 | 0.406 | 1.00 |
| Winter vs Spring | 3.393 | 0.326 | 0.034 | 0.204 | 2.11 | 0.174 | 0.051 | 0.306 |

Values tested were from Euclidian distance matrix that was used for principal component analysis.

P-values were adjusted using the Bonferoni correction for multiple comparisons in the pairwise perMANOVA.

Statistically different pairs (p<0.05) of seasons are italicized and bolded in the Bonferoni Adjusted P-value column.
